# Editorial: Multi-omics assessment for the discovery of promising novel molecules in the treatment of transplant organ injury

**DOI:** 10.3389/fimmu.2025.1744256

**Published:** 2025-11-21

**Authors:** Hao Liu, Xiao-kang Li, Shao-wei Li

**Affiliations:** 1 Department of Surgery, Taizhou Hospital of Zhejiang Province Affiliated to Wenzhou Medical University, Linhai, China; 2 Division of Transplantation Immunology, National Research Institute for Child Health and Development, Tokyo, Japan; 3 Department of Gastroenterology, Taizhou Hospital of Zhejiang Province affiliated to Wenzhou Medical University, Linhai, China

**Keywords:** multi-omics, transplant organ injury, ischemia-reperfusion injury, precision medicine, treatment

Transplant organ injury remains a critical clinical challenge that significantly impacts graft survival and recipient outcomes. The intricate interplay among ischemia-reperfusion injury (IRI), immune rejection, and chronic injury mechanisms highlights the pressing need to identify novel therapeutic molecules (1). The combination of genomics, transcriptomics, proteomics, and metabolomics technologies enables scientists to study organ injury at molecular network levels and discover therapeutic targets. The combination of multiple data types enables researchers to understand individual molecule functions and track how signaling pathways and metabolic changes and immune responses interact with each other to create a complete system for developing personalized treatments (2, 3). The Research Topic showcases modern multi-omics applications for discovering new therapeutic compounds for transplant organ damage through six original and review articles. The research combines human tissue analysis with animal studies to demonstrate how multi-omics approaches speed up the development of new treatments for transplant organ injury through three essential areas of molecular understanding and biomarker discovery and therapeutic target validation.

The core of the review by Wang et al. focused on the applications of single-cell RNA sequencing (scRNA-seq) and spatial transcriptomics (ST) in liver transplantation (LT). The former, through analysis at the single-cell level, revealed the heterogeneity and functional changes of immune cells such as T cells, B cells, and Kupffer cells after transplantation. The latter, leveraging its advantage of preserving tissue spatial structure, compensated for the shortcomings of single-cell sequencing. Meanwhile, they also elaborated on the role of these two technologies in deciphering the mechanisms of liver transplantation immune rejection (FLT3+ dendritic cells inhibit rejection by regulating Tregs) and ischemia-reperfusion injury (IRI) (TNIP3 and MDSC-related signaling pathways alleviate injury).

Similarly, Zhao et al. systematically reviewed the discovery and mechanistic insights into nine key protein post-translational modifications (PTMs), including phosphorylation, ubiquitination, and acetylation. The authors demonstrated that these PTMs initiate and advance hepatic ischemia-reperfusion injury (HIRI) through their effects on cellular signal pathways and oxidative stress and inflammatory reactions and apoptosis and mitochondrial operation. The authors presented essential methods for studying PTMs through mass spectrometry-based analysis and high-throughput screening techniques. The authors identified current research limitations which include studying PTM dynamics in liver microenvironment cells and understanding modification interactions and developing specific biomarkers for real-time monitoring. They demonstrated that PTM intervention strategies show promise for reducing HIRI and enhancing liver transplant results.

The research by Zhou et al. used multi-omics data from transcriptomics and proteomics and metabolomics to study the main damage pathways that occur during ischemia-reperfusion injury (IRI) in liver grafts from donation after circulatory death (DCD). The researchers demonstrated that DCD livers show distinct patterns of gene expression and protein modifications and metabolic patterns when compared to donation after brain death (DBD) livers. Meanwhile, they summarized the emerging therapeutic strategies based on omics findings, including antioxidant drugs, anti-inflammatory interventions, gene editing, metabolic regulation, and combined machine perfusion therapy.

In the field of kidney transplantation, Xiao et al. identified cellular senescence as a characteristic phenotype of renal allograft rejection, particularly T cell-mediated rejection (TCMR), by integrating transcriptomic data from human renal transplant biopsies, mouse and rat transplant models, and single-cell transcriptomic data. They also identified a novel population of stress-induced senescent infiltrating macrophages (SnIMs); these cells possess cross-species conserved transcriptional signatures, are regulated by NF-kB/Cebpb, exhibit cell cycle arrest and upregulation of the senescence-associated secretory phenotype (SASP), and can interact with effector T cells via CXCL chemokines, gradually accumulating during the rejection process. Clinical data showed that SnIM infiltration positively correlates with Banff lesion grades and can predict poor graft survival, thereby providing novel biomarkers and potential targets for the diagnosis and targeted treatment of renal allograft rejection.

To address the issue of insufficient specific data on limb ischemia-reperfusion injury, Shi et al. employed a cross-tissue bioinformatics approach. The researchers combined cardiac IRI transcriptomic data from GSE36073 with rat limb IRI model data to identify four essential genes (WNT5A, PLCG, ITPR1, and CAMK2A) through machine learning methods which they later confirmed to be elevated in limb IRI. The researchers detected immune cell infiltration patterns through their study which showed M1 macrophages and resting dendritic cells rose while M2 macrophages and follicular helper T cells decreased. The research established connections between core genes and particular immune cell populations. The WNT5A/PLC pathway became a therapeutic target after researchers proved its role in tissue protection through pharmacological blocking of this pathway. The research establishes a new method to study limb IRI mechanisms and develop treatment approaches.

The review by Lian et al. examined how ischemia-reperfusion injury (IRI) affects intestinal microecology in organ transplantation and its therapeutic applications. The authors demonstrated that IRI damages intestinal mechanical and chemical and immune and biological barriers which results in gut microbiota dysbiosis. The disruption of gut microbiota composition makes patients more susceptible to post-transplant infections and acute rejection episodes which negatively impacts graft survival. The therapeutic potential of microbial-based interventions including probiotics and prebiotics and fecal microbiota transplantation and bacteriophage therapy shows promise for enhancing transplant results by repairing intestinal equilibrium which guides medical practice and scientific investigation.

The current development of multi-omics technologies enables scientists to shift organ injury treatment in transplanted organs from random approaches to specific precision medicine methods. The research requires two main steps to achieve its goals which include solving current data integration problems and building analytical systems that reveal molecular network causal relationships. The research needs to expand its collection of clinical multi-omics data while working to validate biomarkers and therapeutic targets for clinical use. The combination of single-cell multi-omics with spatial omics and artificial intelligence algorithms will create a foundation for developing personalized treatment plans for each individual patient. The research findings from this study will create new knowledge for laboratory work and medical practice which will help develop precise transplant medical treatments.

## References

[1] Liu X Shen J Yan H Hu J Liao G Liu D . Posttransplant complications: molecular mechanisms and therapeutic interventions. MedComm (2020). (2024) 5:e669. doi: 10.1002/mco2.669, PMID: 39224537 PMC11366828

[2] Robertson H Kim HJ Li J Robertson N Robertson P Jimenez-Vera E . Decoding the hallmarks of allograft dysfunction with a comprehensive pan-organ transcriptomic atlas. Nat Med. (2024) 30:3748–57. doi: 10.1038/s41591-024-03030-6, PMID: 38890530 PMC11645273

[3] Loupy A Preka E Chen X Wang H He J Zhang K . Reshaping transplantation with AI, emerging technologies and xenotransplantation. Nat Med. (2025) 31:2161–73. doi: 10.1038/s41591-025-03801-9, PMID: 40659768

